# Integration of open access literature into the RCSB Protein Data Bank using BioLit

**DOI:** 10.1186/1471-2105-11-220

**Published:** 2010-04-29

**Authors:** Andreas Prlić, Marco A Martinez, Dimitris Dimitropoulos, Bojan Beran, Benjamin T Yukich, Peter W Rose, Philip E Bourne, J Lynn Fink

**Affiliations:** 1 San Diego Supercomputer Center, University of California San Diego, 9500 Gilman Drive, Mailcode 0505 La Jolla, CA 92093-0505 USA; 2 Skaggs School of Pharmacy and Pharmaceutical Sciences, University of California San Diego, 9500 Gilman Drive, Mailcode 0743, La Jolla, CA, 92093-0743 USA

## Abstract

**Background:**

Biological data have traditionally been stored and made publicly available through a variety of on-line databases, whereas biological knowledge has traditionally been found in the printed literature. With journals now on-line and providing an increasing amount of open access content, often free of copyright restriction, this distinction between database and literature is blurring. To exploit this opportunity we present the integration of open access literature with the RCSB Protein Data Bank (PDB).

**Results:**

BioLit provides an enhanced view of articles with markup of semantic data and links to biological databases, based on the content of the article. For example, words matching to existing biological ontologies are highlighted and database identifiers are linked to their database of origin. Among other functions, it identifies PDB IDs that are mentioned in the open access literature, by parsing the full text for all research articles in PubMed Central (PMC) and exposing the results as simple XML Web Services. Here, we integrate BioLit results with the RCSB PDB website by using these services to find PDB IDs that are mentioned in research articles and subsequently retrieving abstract, figures, and text excerpts for those articles. A new RCSB PDB literature view permits browsing through the figures and abstracts of the articles that mention a given structure. The BioLit Web Services that are providing the underlying data are publicly accessible. A client library is provided that supports querying these services (Java).

**Conclusions:**

The integration between literature and websites, as demonstrated here with the RCSB PDB, provides a broader view for how a given structure has been analyzed and used. This approach detects the mention of a PDB structure even if it is not formally cited in the paper. Other structures related through the same literature references can also be identified, possibly providing new scientific insight. To our knowledge this is the first time that database and literature have been integrated in this way and it speaks to the opportunities afforded by open and free access to both database and literature content.

## Background

Biological databases and the biological literature have traditionally been distinct resources. The main difference being that databases are providing structured information, while articles are essentially free text and unstructured. As we have argued in the past [1-3], this is an artificial distinction, in part defined by the way each resource is perceived, and in part because databases are an on-line medium and journals have traditionally been hardcopy. As the scientific literature has moved on-line, the distinction has blurred. Databases have become more like the literature by including an increased amount of annotation, often extracted from the literature [4]. Conversely, the literature has become more like databases by including an increasing amount of supplemental information, including the data derived from the experiments described in the research article.

The blurring of the distinction between the biological literature and biological databases is furthered by open access that (depending on the specific open access license) provides literature in a free and unrestricted XML marked-up form as defined by the National Library of Medicine (NLM) Document Type Definition (DTD). By parsing the literature available in this XML form it is possible to extract semantic meaning. We have taken a relatively simple approach to this by extracting semantics associated with database identifiers and ontology terms [5]. With these terms, which are typically already well defined in a variety of biological databases, it is possible to create interesting associations between database and literature content.

We illustrate this here by integrating the content of PubMed Central (PMC) with that of the RCSB Protein Data Bank (PDB) [6] via the BioLit [5] resource. At this time only about 21% of structures in the PDB (as defined by their PDB identifiers) are referenced in the full text of open access articles contained in PMC, but already some interesting associations can be made. The immediate impact when viewed from the RCSB PDB is that new literature references to the structure are uncovered, even when the primary citation to the structure (if one exists) is not cited in that literature. As described subsequently, appropriate components of that literature can then be integrated with database content and presented as a unified view; a small step towards true literature-data integration.

Many groups have made significant contributions in extracting semantic data from both open- and closed-access literature in the life sciences, although none focus on PDB IDs. In particular, GoPubMed [7], SEGOPubmed [8], and Textpresso [9], all web resources, have improved the classification and searchability of articles by inferring semantic relationships in articles using existing or customized ontologies [9,10]. In structural biology, PDBsum [11] has gained permission from publishers to use selected figures and captions for the PDBsum pages and the figures are extracted from the journal's websites using custom made scripts. The FEBS Letters engaged a collaboration with the MINT database aimed at integrating each manuscript with a structured summary, which precisely reports the protein interactions reported in the manuscript. This is achieved using database identifiers and predefined controlled vocabularies [12].

We proceed by describing the methods used to establish RCSB PDB-BioLit integration, followed by examples of how that integration is presented and conclude with future directions afforded by free and unrestricted access to database and literature content.

## Implementation

### BioLit pipeline

BioLit is a resource that delivers semantically enriched content for all research articles from PubMed Central (PMC) [5]. Specifically, this content includes database identifiers and ontology terms found within the full text of the articles. BioLit uses a local copy of PMC and applies a text-mining pipeline in order to identify ontology terms provided by a number of ontologies from the National Center for Biomedical Ontology (NCBO, http://bioportal.bioontology.org/). If matches to multiple terms are found BioLit identifies and applies the longest possible match. In addition BioLit identifies PDB IDs in the articles. The IDs are identified by pattern matching which includes heuristics to avoid false positives. A validity check is performed by comparing possible matches with existing PDB IDs. BioLit provides weekly updates from PMC using a cron job to fetch the latest articles (approximately 1,000-2,000 per week at the time of writing).

### Web Service Communication between BioLit and RCSB PDB

The BioLit web and database servers are independent of the servers that are hosting the RCSB PDB site. For integration between the two resources RESTful Web Services are used to communicate XML documents over HTTP. Two example URLs are given to demonstrate the communication process: The first URL allows access to BioLit information for all articles containing a particular PDB ID: http://biolit.ucsd.edu/ws/rest/articles/pdbid/1HIV/metadata (to request articles for PDB ID 1HIV). The second URL allows access to descriptions of the Figures in a specific article, based on its PMC ID: http://biolit.ucsd.edu/ws/rest/files/pmcid/1483839/figures. For more detailed documentation of these Web Services, see the online documentation at http://biolit.ucsd.edu/doc/rest.jsp. To simplify communication with these services and in order to allow other people to access these data, we provide a simple Web Service client (Java). This client library is used by the RCSB PDB web site to request data from the BioLit servers. The data are then rendered in a user's browser using standard Javascript and JSON web technologies.

To make sure the latest literature associations are available to RCSB PDB users, the data are requested dynamically from BioLit prior to visualization on the website. However, to provide a fast response once a given PDB structure is requested, results are cached RCSB PDB-server side using the Memcached library http://www.danga.com/memcached.

## Results

### PDB Integration

At present, BioLit has identified articles in PMC for approximately 21% of PDB structures (at this time 13,273 PDB IDs). Currently 44,984 articles in the BioLit copy of PMC mention PDB IDs.

**Figure 1:**
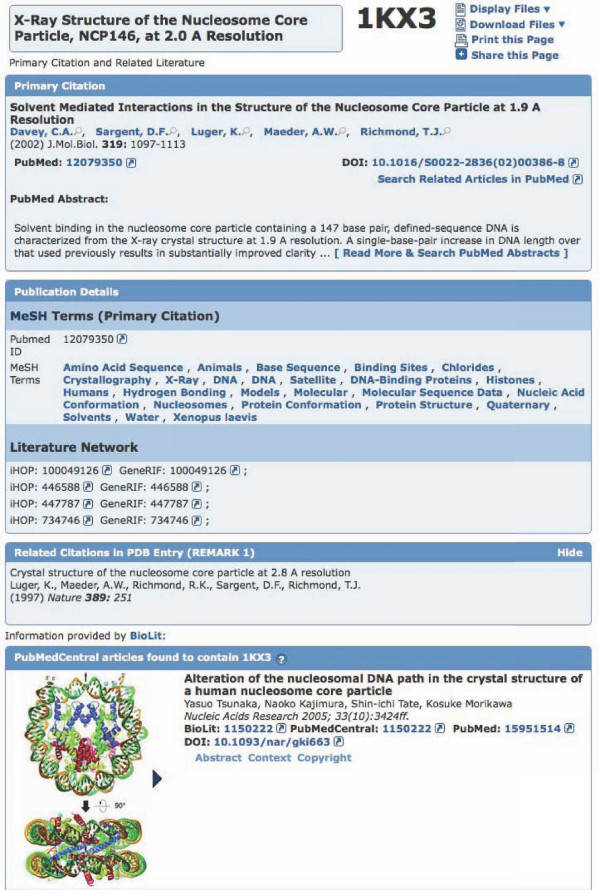
**Literature tab view for PDB ID **1KX3**from the RCSB PDB**. The view provides the following data fields: *Primary Citation *for the protein structure; *Publication Details*: MeSH Keywords for the article and related citations from iHOP and GeneRIF. *Related Citations in PDB entry *as provided by the depositors of the structure; *PubMed Central articles *are articles identified by BioLit that mention the PDB ID; *Other PDB IDs *(not shown) that co-occur with 1KX3 in PubMedCentral articles.

If articles are available from BioLit, the new "Literature" tab on the structure summary page of the RCSB PDB website provides a comprehensive summary of the associated literature. An example of how this is displayed is given in Figure 1.

The "Literature" tab provides several sections of information (if available) for a given PDB structure:

1. *Primary Citation *presents the abstract and literature reference information for the original article associated with the PDB structure.

2. *Publication Details *displays MeSH Keywords for the article and links to related citations from the iHOP [13] and GeneRIF sites [14].

3. *Related Citations *show secondary literature references provided by the depositors of the structure.

4. *PubMed Central articles *displays articles that have been identified by BioLit to contain references to the PDB structure. In this section of the web page it is possible to browse through all the figures provided in the original article. If a thumbnail is selected, a larger version of the figure as well as the figure legend is displayed. Additionally, the article abstract and copyright information can be displayed. The *Context *in which a PDB ID has been mentioned in an article can be investigated as well. Usually this will be the surrounding text paragraph. In the case of figures it displays the figure legend. All content is made available under the same copyright that applies to the original material.

5. *Other PDB IDs *that are found in the same articles as the target PDB ID are also listed. This list provides the user with an additional set of structures that are referenced in the same paper(s). Structures grouped by the same literature references may or may not indicate some common features. In order to provide further information on what it means if two entries are cited in the same article, we also show the sequence similarity between the co-occurring IDs. In cases where a PDB structure contains multiple chains the one with the highest similarity is displayed.

### Finding Citations that are not in the Article Reference List

The major value of the literature and database integration is to identify citations to articles that are not in the article reference list. For example, the PDB ID 3BY7 was cited by an article in Genome Biology [15] even before the primary citation for the protein structure was published [16]. The identification of such citations would be impossible without PMC and subsequently BioLit.

## Conclusions

At present research articles for approximately 21% of the structures in the Protein Data Bank (PDB) are found in PubMed Central (PMC). This number is expected to increase rapidly given that many of the world's research funding agencies have specified that the publications associated with the research they fund must be made open access and hence available through PMC. For example the NIH stipulates that research publications they fund be accessible within one year of publication http://publicaccess.nih.gov/.

One of the difficulties when data mining PDB IDs is that at 4-characters in length they may not be unique. For example, the PDB ID 3DNA had several false positive hits that were found to refer to a software package and a wet lab kit, both sharing the name "3DNA." By applying contextual criteria within the text mining and data analysis pipeline such misleading results can be filtered. Still subsequent manual review is the only method for achieving total accuracy. We are maintaining a list of PDB IDs that are known to contain false positive hits and for which stricter criteria are applied. This simple approach has turned out to work well to filter out false positive articles.

The future calls for unique identifiers. Digital Object Identifiers (DOIs) are already provided for each PDB structure, in a manner similar to research articles, so the authoritative reference to the content associated with the DOI can be resolved in an Internet environment. This is a positive step, but at present few research articles provide DOIs to reference structures. Moreover, this does not resolve the finer parts of a macromolecular structure, for example individual polypeptide chains and ligands, each of which are often referenced specifically in research articles.

We describe an initial step in database and literature integration using common and reliable semantics to create the linkage between the two previously disparate resources. The intent is to show the promise that this approach has to providing improved comprehension, not just to users of the RCSB PDB, but if implemented by other databases, to those as well. Semantic association through database identifiers is just a first step in what is possible as PMC increases in content. Ontological terms commonly used by databases can be located in the literature and richer and more contextual interrelationships are possible. In the case of the RCSB PDB a sign of success would be for the user to identify from literature integration a function of the protein previously unknown to them. Tool development to hopefully facilitate this type of discovery is on-going.

### Availability and requirements

#### Most Frequently Cited PDB Structures

Table 1 lists the eight most frequently cited PDB structures in PMC and their associated functions. A longer-term goal is to extract functional associations automatically from PMC.

**Table 1:** Structures appearing most frequently in PubMed Central (PMC), based on the citations identified using the BioLit pipeline

PDB ID	Protein Name	Nr. of Articles
1JJ2	Large Ribosomal Subunit	27

1J5E	30S Ribosomal Subunit	19

1FFK	Large Ribosomal Subunit	19

1LMB	Lambda Repressor	19

1AAY	Zinc Finger	17

1TSR	P53	16

1F88	Rhodopsin	15

1BRS	Barnase/Barstar complex	14

The open access literature for RCSB PDB entries is available from the Literature tab for each structure entry at http://www.rcsb.org. The RESTful Web Services provided by BioLit http://biolit.ucsd.edu are documented at http://biolit.ucsd.edu/doc/rest.jsp. The client library is written in Java to simplify communication with these services can be downloaded at http://biojava.org/wiki/BioLit.

## Authors' contributions

AP wrote the BioLit client library, provided the new literature view and drafted the manuscript. MAM & JLF developed BioLit and provided the Web Services. MAM maintains updates of PMC and implemented the false positive filters. BTY and DD provide development and support of the servers. BB contributed to the development of the literature view. PR participated in the design and coordination. PEB conceived of the idea, directed the research and wrote sections of the manuscript. JLF coordinated and managed the BioLit project and helped draft the manuscript. All authors read and approved the final manuscript.
